# Vitamin D Status and Association of *VDR* Genetic Polymorphism to Risk of Breast Cancer in Ethiopia

**DOI:** 10.3390/nu11020289

**Published:** 2019-01-29

**Authors:** Jemal Hussien Ahmed, Eyasu Makonnen, Alan Fotoohi, Getnet Yimer, Daniel Seifu, Mathewos Assefa, Wondmagegnehu Tigeneh, Abraham Aseffa, Rawleigh Howe, Eleni Aklillu

**Affiliations:** 1 Department of Pharmacology and Clinical Pharmacy, Addis Ababa University, P.O. Box 9086 Addis Ababa, Ethiopia; eyasumakonnen@yahoo.com (E.M.); getnetyimer@yahoo.com (G.Y.); 2 Department of Pharmacy, Jimma University, P.O. Box 378 Jimma, Ethiopia; 3 Division of Clinical Pharmacology, Department of Laboratory Medicine, Karolinska Institutet, Karolinska University Hospital, Huddinge, 141 86 Stockholm, Sweden; Alan.Fotoohi@ki.se (A.F.); Eleni.Aklillu@ki.se (E.A.); 4 Center for Innovative Drug Development and Therapeutic Trials, Addis Ababa University, P.O. Box 9086 Addis Ababa, Ethiopia; 5 Division of Clinical Pharmacology, Department of Medicine, Karolinska Institutet, 171 76 Solna, Stockholm, Sweden; 6 Ohio State Global One Health initiative, Office of international affairs, Ohio State University, P.O. Box 9842 Addis Ababa, Ethiopia; 7 Department of Biochemistry, Addis Ababa University, P.O. Box 9086 Addis Ababa, Ethiopia; daniel.seifu@aau.edu.et; 8 Radiotherapy center, Addis Ababa University, P.O. Box 9086 Addis Ababa, Ethiopia; mathewosassefa80@hotmail.com (M.A.); Tigeneh@yahoo.com (W.T.); 9 Armauer Hansen Research Institute, P.O. Box 1005 Addis Ababa, Ethiopia; aseffaa@gmail.com (A.A.); rawcraig@yahoo.com (R.H.)

**Keywords:** vitamin D deficiency, *VDR*, genetic variations, breast cancer, Ethiopia

## Abstract

Emerging evidence associates vitamin D deficiency and vitamin D receptor (*VDR*) genetic variations with risk for breast cancer. This study investigated the prevalence of vitamin D deficiency and its association with tumor characteristics and the implications of *VDR* genetic variations for risk of breast cancer in Ethiopia. This unmatched case–control study involved 392 female breast cancer patients and 193 controls. The plasma 25-hydroxyvitamin D (25(OH)D_3_) level was quantified in chemotherapy-naïve (*N* = 112) and tamoxifen-treated patients (*N* = 89). Genotyping for the *VDR* common variant alleles rs7975232 (*Apa*I), rs2228570 (*Fok*I), and rs731236 (*Taq*I) was done. Eighty-six percent of the patients were vitamin D deficient (<50 nmol/L). Chemotherapy-naïve breast cancer patients had a higher prevalence of vitamin D deficiency (91.9% vs. 78.3%) compared to the tamoxifen-treated group (*p* < 0.001). The prevalence of severe vitamin D deficiency (<25 nmol/L) was significantly higher in chemotherapy-naïve (41.1%) than tamoxifen-treated (11.2%) patients. Vitamin D deficiency was not significantly associated with tumor characteristics or *VDR* genotype. The rs2228570 *GG* genotype was associated with increased risk of breast cancer (OR = 1.44, 95% confidence interval = 1.01−2.06). Our result indicates that rs2228570 might be a moderate risk factor for breast cancer development in the Ethiopian population. The high prevalence of severe vitamin D deficiency in treatment-naïve breast cancer patients indicates the need for nutritional supplementation of vitamin D at the time of chemotherapy initiation.

## 1. Introduction

Vitamin D has long been known for its physiological role in calcium balance and for being responsible for increased intestinal absorption of calcium, magnesium, and phosphate [1]. It plays a crucial role in the proper functioning of the immune, muscle, and nervous systems [2,3], as well as in controlling normal cell growth [2]. In addition, vitamin D is reported to have anticancer activities against many cancer types, including breast cancer [4]. Reports from epidemiologic [5,6] and mechanistic studies [7] have demonstrated that vitamin D inhibits cancer cell proliferation, induces apoptosis, and decreases angiogenesis. 

In humans, most of the vitamin D is generated from sunlight-mediated conversion of dehydrocholesterol in skin to form cholecalciferol (Vit D_3_). As Vit D_3_ has little biological activity, it undergoes two hydroxylation reactions to become biologically active. The first hydroxylation occurs in the liver, catalyzed by several Cytochrome P450 enzymes, such as *CYP2R1, CYP27A1, CYP2D25*, and *CYP2J3*, and gives rise to 25-hydroxyvitamin D_3_ (25(OH)D_3_). A further hydroxylation in the kidney by the action of *CYP27B1* converts 25(OH)D_3_ to 1,25-dihydroxyvitamin D_3_ (1,25-(OH)_2_D_3_, calcitriol), the hormonally active form of vitamin D [8]. The bioactive 1,25-(OH)_2_D_3_ functions by binding to a nuclear vitamin D receptor (*VDR*) to regulate gene transcription [9]. Due to its abundance and longer plasma half-life, 25(OH)D_3_ concentration is the parameter of choice for the assessment of vitamin D status [10].

A number of variables, including latitude, season, time of day, atmospheric components, clothing, sun screen use, and skin pigmentation influence the amount of UVB radiation entering the skin, thereby affecting vitamin D production. Moreover, other factors such as age, gender, physical activity, and obesity, as well as chronic illnesses such as cancer, could affect the synthesis and bioavailability of vitamin D [10,11]. It has become evident that vitamin D deficiency is a major problem in many populations worldwide [12]. Although high concentrations of vitamin D have been observed in some East African ethnic groups [13,14], low serum 25(OH)D_3_ levels were reported in many African populations, including Algerian pregnant women and Egyptian healthy children [15]. Similarly, high rates of vitamin D deficiency or insufficiency has been reported in Ugandan children [16] and adult patients with human immunodeficiency virus (HIV) and tuberculosis (TB) [17,18]. Previous studies in Ethiopia also showed a high prevalence of low vitamin D in men and non-pregnant women [19], school children [20], and TB/HIV co-infected people [21].

The implications of vitamin D deficiency on cancer susceptibility have been demonstrated in previous studies. Association between a low serum 25(OH)D_3_ level and increased breast cancer development, risk for breast cancer recurrence, and mortality has been reported [22]. A recent meta-analysis demonstrated a protective relationship between high circulating 25(OH)D_3_ level and breast cancer development in premenopausal women [23]. It was also shown that, in patients with 25(OH)D_3_ level below 50 nmol/L, there is a higher predicted probability of breast cancer among African Americans compared to Hispanics [24]. A pooled analysis of randomized trials demonstrated that higher 25(OH)D_3_ concentrations were associated with a decrease in breast cancer risk, with concentrations of ≥ 150 nmol/L being most protective [25]. 

Genetic variations in the *VDR* genes have also been associated to risk of breast cancer [26,27,28,29]. The link between *VDR* genetic variations and breast cancer risk is attributed to the notion that the action of vitamin D is mediated by *VDR.* In addition, both normal and breast cancer cells have been found to express the receptor [30]. The *VDR* genetic variation is also associated with better survival benefit [29,31]. The *VDR* variant alleles which have been studied include rs7975232 (A > C, commonly known as *ApaI*), rs1544410 (T > C, commonly known as *Bsm*I), rs2228570 (T > C, commonly known as *Fok*I), and rs731236 (T > C, commonly known as *Taq*I). However, reports regarding the specific relevance of *VDR* allelic variations on breast cancer risk have been inconsistent. An increased risk of breast cancer was observed with the rs1544410 *bb* or *Bb* genotype, but not with the rs2228570 genotype in Iranian [32] and Egyptian [33] female breast cancer patients. Another research finding demonstrated that, compared with homozygotes for the common rs2228570 *F* allele (*FF* genotype), *ff* homozygotes had a higher breast cancer risk [34]. On the other hand, a population-based cohort study showed no association of single-nucleotide polymorphisms (SNPs) in the *VDR* with cancer incidence [35]. Associations were also not detected between breast cancer risk and genotype and allele frequencies of rs2228570 and rs731236 polymorphisms [36]. 

These discrepancies suggest the need for further exploration of the issue from various population groups. Furthermore, the prevalence of vitamin D deficiency and type of breast cancer varies between populations. Black African populations display large genetic diversity compared to Asians and Caucasians. On the other hand, information regarding the prevalence of vitamin D deficiency or insufficiency in cancer patients in the black African population is unavailable. Although abundant sunshine is available, the population is reported to be at risk of low vitamin D levels due to insufficient sun exposure associated with socio-cultural factors [19]. As most cancer patients are more likely to spend most of their time indoors, we hypothesize that cancer patients could be at a greater risk of low vitamin D levels. The genetic constitution of Ethiopians could also add pertinent information related to the role of *VDR* gene polymorphism in breast cancer susceptibility. Thus, the aim of the current study was to examine the prevalence and severity of vitamin D deficiency and the association of *VDR* polymorphism with risk of breast cancer in Ethiopia.

## 2. Materials and Methods 

The study was conducted at the radiotherapy center (9° N, 38° E) of Tikur Anbessa Specialized Hospital (TASH), Addis Ababa, Ethiopia. An unmatched case–control study design was employed involving breast cancer patients (cases) and control groups from the general population with no history of breast cancer at the time of study enrolment (controls). The inclusion criteria for cases were adult female patients with pathologically and clinically diagnosed breast cancer. All consecutive and volunteer patients (stages I–IV) who came for the first cycle of the chemotherapy regimen (treatment/chemotherapy naïve) or those who completed their chemotherapy and were on tamoxifen adjuvant therapy (tamoxifen group) in the outpatient day-care ward of TASH were included. Controls were recruited from the general population from TASH. Exclusion criteria for participants (cases) were a prior history of vitamin D supplementation (for vitamin D assay only), pregnant or breastfeeding women, and previous neo-adjuvant chemotherapy.

The study protocol was approved by Armauer Hansen Research Institute Ethical Review Committee (AAERC) (Ref No: PO26/16), Institutional Review Board (IRB) of the College of Health Sciences, Addis Ababa University (Ref No: 011/16/2016), and National Research Ethics Review Committee (NRERC) of the Federal Democratic Republic of Ethiopia (Ref No: 3.10/235/2017). Signed informed consent was obtained from individual patients prior to participation in the study. 

Patients’ baseline medical records, laboratory investigations (blood counts and organ function estimates), and results of biopsy reports describing tumor characteristics such as site of tumor, degree of differentiation, tumor size, and lymph node involvement were recorded. In addition, menopausal status, Karnofsky’s performance status (performance scale > 70), weight, height, body mass index (BMI), chemotherapy panel (for chemotherapy groups either as neo-adjuvant, adjuvant, or metastatic), and chemotherapy regimen were recorded. 

The sample size required for a 95% two-sided confidence interval for an unmatched case–control study with 90% power to detect a risk ratio of 2 for breast cancer (associated with *VDR* genetic polymorphism) was calculated using OpenEpi^R^ software [37]. Accordingly, considering a case-to-control ratio of 2 (2 cases:1 control) (*n* = 548) and a 5% addition, the estimated sample size (*N*) was 575 (190 controls vs. 383 cases).

### 2.1. Genotyping 

Whole blood samples were collected in EDTA tubes from all participants and genomic DNA was isolated using a QIAamp DNA Midi Kit (Qiagen GmbH, Hilden, Germany). Genotyping for the common functional variant alleles of *VDR* genes relevant to breast cancer risk—*Fok*I, *Apa*I, and *Taq*I—was carried out using Taqman allele-specific PCR (Applied Biosystems Genotyping Assays) as described previously [38]. In brief, genotyping was performed using TaqMan^®^ SNP genotyping assay reagents for allelic discrimination (Applied Biosystems, Waltham, MA, USA) with the following ID number for each SNP: C__12060045_20 (rs2228570, T > C), C__2404008_10 (rs731236, T > C), andC__28977635_10 (rs7975232, A > C). Genotyping was carried out using a QuantStudio 12K Flex Real-Time PCR system (Life Technologies Holding, Singapore). The final volume for each reaction was 10 μL, consisting of TaqMan^®^ fast advanced master mix (Applied Biosystems, Waltham, MA, USA), TaqMan 40X SNP genotyping assays mix (Applied Biosystems, Waltham, MA, USA), and genomic DNA. The PCR parameter consisted of an initial step at 60 °C for 30 s, hold stage at 95 °C for 10 min, PCR stage for 40 cycles: Step 1 at 95 °C for 15 mins and Step 2 at 60 °C for 1 min, and a read stage after at 60 °C for 30 s. Genotypes were assigned using the manual calling option in the allelic discrimination application, using QuantStudio 12K Flex software (Applied Biosystems, Life Technologies, Stockholm, Sweden). The characterized SNPs were selected on the basis of their potential to influence the functionality of the vitamin D receptor, as obtained from public databases. The genomic DNA of the known genotype and two no template controls (NTCs) were run in parallel to the samples.

### 2.2. Vitamin D Quantification

The plasma vitamin D level was measured from a total of 201 breast cancer patients, of which 112 were treatment naïve (planned to initiate their first cycle chemotherapy) and 89 were on tamoxifen at 20 mg/day, following completion of their course of chemotherapy. The plasma vitamin D level was quantified using DiaSorin assay at the Centre for Chemical Laboratory, Karolinska University Laboratory, Solna, Sweden. Plasma vitamin D status was classified according to the recommended guideline [39]. Accordingly, severe vitamin D deficiency (SVDD) and vitamin D deficiency (VDD) were defined as circulating 25(OH)D_3_ levels of < 25 nmol/L, and 25–50 nmol/L, respectively. Levels between 51 and 72.5 nmol/L were considered insufficient [21]. 

### 2.3. Statistical Analysis

Descriptive analyses are presented for the demographic and clinical characteristics.The genotype and allele frequencies were assessed by Chi-square test to compare observed and expected genotypic distributions. Nonparametric testing was used to compare plasma 25(OH)D_3_ levels between chemotherapy-naïve and tamoxifen groups. One-way ANOVA was used to test the relationship between vitamin D concentration and genotype. The association between breast cancer and *VDR* polymorphisms were assessed first with χ^2^ test, and then the odds ratio was estimated using logistic regression models to see the magnitude of association. The data were analyzed using SPSS software version 21.0 for Windows (IBM Corporation, NY). A *p*-value of < 0.05 was considered statistically significant for each test, and Bonferroni correction (as the number of hypotheses is fairly small) was then applied for multiple comparisons. Graphs were prepared using GraphPad Prism software, v.7.04 (GraphPad Software, La Jolla, CA, USA).

## 3. Results

**Figure 1:**
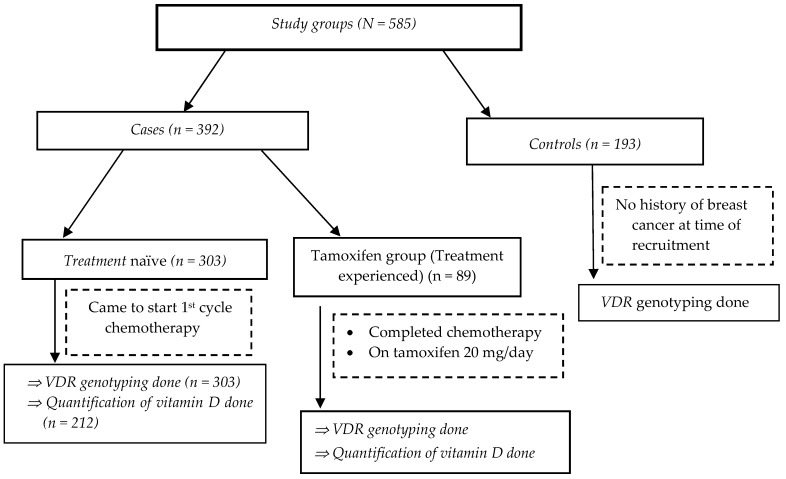
Study flow chart depicting study groups and participant recruitment.

A total of 585 study participants were enrolled, comprising 392 breast cancer cases and 193 controls. The study flow chart and recruitment procedure is depicted in Figure 1. Out of the breast cancer cases (*n* = 392), 303 were chemotherapy-naïve patients who came for their first cycle of chemotherapy, and 89 were treatment-experienced patients who had completed the full course of chemotherapy and were on tamoxifen therapy. The baseline socio-demographic, clinical, and laboratory parameters and tumor profiles of study participants are presented in Table 1. The common chemotherapy regimens planned for the chemotherapy-naïve patients (*n* = 392) were FAC (5-**F**lourouracil 500 mg/m^2^, **A**driamycin [Doxorubicin] 50 mg/m^2^, and **C**yclophosphamide 500 mg/m^2^) (41.3%), AC – T (**A**driamycin 60 mg/m^2^ and **C**yclophosphamide 600 mg/m^2^ followed by Taxol 175 mg/m^2^) (39.3%), and AC (**A**driamycin 50 mg/m^2^ and **C**yclophosphamide 600 mg/m^2^) (18.2%).

### 3.1. Association of VDR Polymorphism and Breast Cancer Risk

The genotype distribution (Table 2) indicated that all the *VDR* genes in the case and control groups conformed to the Hardy–Weinberg equilibrium (HWE) (*p*-value > 0.05). The overall frequencies of alleles for *rs7975232* (allele C), *rs2228570* (allele A), and *rs731236* (allele G) in Ethiopians were 0.39, 0.21, and 0.38, respectively. There was no significant difference in genotype and allele distribution between breast cancer patients (cases) and control groups (*p* > 0.05) (Table 2) except for a trend (*p* = 0.078) showing a higher frequency of the *rs2228570 G* (*F*) variant allele in cases than in controls. 

Stratifying by variant allele carrier status, the *rs2228570 GG* (*GG* vs. *AA* + *AG*) genotype was found to be associated with breast cancer risk (OR = 1.44, 95% confidence interval = 1.01–2.05) (Table 3). The presence of this genotype could confer a 44% increase in the risk of acquiring breast cancer. However, no association was detected between homozygotes for the common *rs2228570 F* allele (*GG* genotype) and *ff* homozygotes (*p* > 0.05). Similarly, *rs7975232* (*CC vs. AA* + *AC* and *C* vs. *A*) and *rs731236* (*GG* vs. *AA* + *AG* and *G* vs. *A*) polymorphisms were not associated with the risk of breast cancer.

### 3.2. Plasma 25(OH)D_3_ Concentration and Vitamin D Status among Treatment-Naïve Versus Tamoxifen-Treated Patients

**Figure 2:**
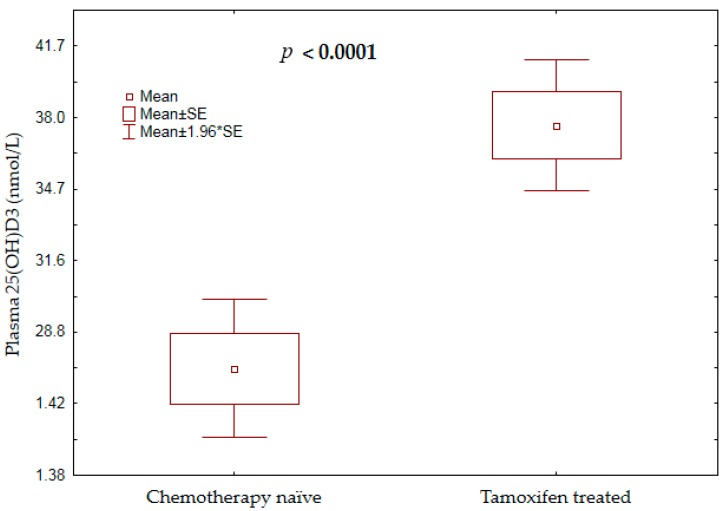
Comparison of mean plasma 25(OH)D_3_ concentrations between chemotherapy-naïve patients and those on tamoxifen treatment.

The study outcome variables were plasma 25(OH)D_3_ level and vitamin D status; vitamin D deficiency (VDD) was defined as circulating levels of 25(OH)D_3_ of < 50  nmol/L, and levels between 51 and 72.5  nmol/L were considered insufficient. Severe vitamin D deficiency (SVDD) was defined as a plasma 25(OH)D_3_ level of < 25  nmol/L [21]. The mean plasma 25(OH)D_3_ concentration among chemotherapy-naïve patients was significantly lower compared to that among the patients on tamoxifen (*p*-value < 0.0001, Figure 2). 

A comparison of Vitamin D Status between chemotherapy-naïve and treatment-experienced patients (tamoxifen group) is presented in Table 4. Overall, 86% of the studied patients were vitamin D deficient with 28% being SVDD and 58% VDD (Figure 1). In the tamoxifen group, the prevalence of vitamin D deficiency was 78.3%, with SVDD and VDD accounting for 11.2% and 67.4%, respectively. On the other hand, 91.9% of chemotherapy-naïve breast cancer patients were vitamin D deficient (Table 4). 

### 3.3. Vitamin D Status and Tumor Characteristics

Breast cancer characteristics (degree of differentiation, tumor size, lymph node involvement, or distant metastasis to liver or lung) were not significantly associated with vitamin D deficiency (*p*-value > 0.05, Chi-square test). 

### 3.4. VDR Genotype with Plasma 25(OH)D_3_ Concentration and Vitamin D Status 

**Figure 3:**
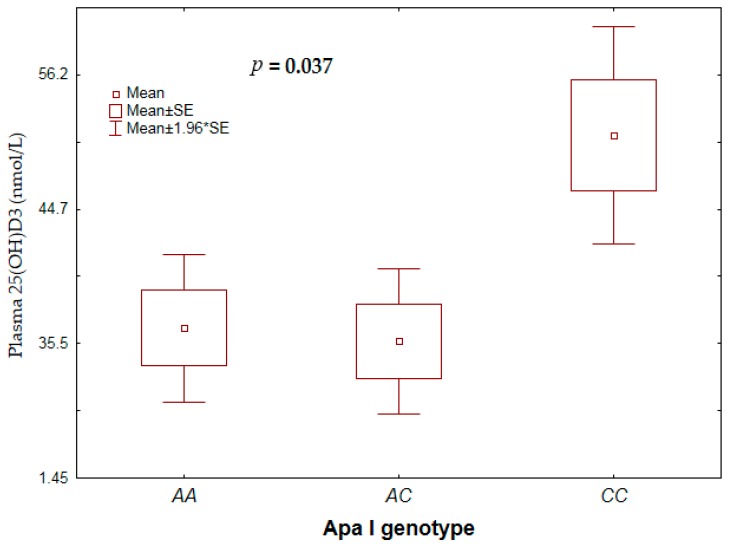
Association between vitamin D deficiency status and rs7975232 (*Apa*I) genotype.

Overall, there was no significant influence of *VDR* genotype on plasma 25(OH)D_3_ concentration. However, stratified by treatment group, the rs7975232 (*Apa*I) *CC* genotype was significantly associated with higher plasma 25(OH)D_3_ concentration than the *AA* or *AC* genotype among the tamoxifen group (Figure 3). No such association was found in chemotherapy-naïve patients. There was also no significant association between vitamin D deficiency status and genotype or allele frequency (*p*-value > 0.05) 

## 4. Discussion

In recent years, several studies focusing on the effect of the vitamin D pathway have been carried out, mainly in Europeans and African Americans, and the results have generally showed the importance of vitamin D and *VDR* genetic variations in a number of clinical conditions. To the best of our knowledge, there are no published data on vitamin D status among chemotherapy-naïve and treatment-experienced breast cancer patients or on the impact of *VDR* gene polymorphisms on risk for development of breast cancer in the Sub-Saharan black African population, where there is abundant sunshine to form vitamin D. 

In the present study, we have observed that vitamin D deficiency is rampant among Ethiopian breast cancer patients, with 86% of them either severely vitamin D deficient (28%) or vitamin D deficient (58%). Chemotherapy-naïve breast cancer patients had significantly lower mean vitamin D levels compared to those of the tamoxifen-treated group (Figure 2). Likewise, the prevalence of severe vitamin D deficiency was much higher among treatment-naïve patients (41%) than among those who survived to complete chemotherapy and continue long-term tamoxifen adjuvant therapy (11%). Although not proven, the results of previous studies showed that plasma vitamin D concentration could influence survival outcomes in cancer patients [40,41,42]. From this perspective, the studied patients may benefit from increasing their vitamin D levels through vitamin D supplementation, particularly in chemotherapy-naïve patients. 

On the other hand, evidence on vitamin D level relative to cancer occurrence is inconclusive. In patients with a 25(OH)D_3_ level below 50 nmol/L, higher predicted probabilities of breast cancer have been reported among African-Americans compared to Hispanics [24]. A result of pooled analysis of two randomized trials and a prospective cohort study demonstrated that breast cancer risk was markedly lower with a higher circulating 25(OH)D_3_ level (≥150 nmol/L) [25]. Similarly, the result of a meta-analysis showed a protective relationship between circulating 25(OH)D_3_ level and breast cancer development in premenopausal women [23]. On the other hand, a recent nationwide, randomized, placebo-controlled trial (VITAL trial), concluded that supplementation with vitamin D did not result in a lower incidence of invasive cancer or cardiovascular events [43].

Vitamin D deficiency has been described as a problem in many countries around the world, including in Africa [12]. Vitamin D status depends largely on the production of vitamin D_3_ in the skin under the influence of ultraviolet radiation and, to some extent, on vitamin D intake through the diet or vitamin D supplements [12]. Vitamin D status could also be associated with underlying health conditions and treatment modality [21,44]. Consequently, vitamin D production varies considerably around the world, across population groups, and between individuals, mainly because of wide differences in skin exposure to ultraviolet B (UVB) radiation, efficiency of cutaneous synthesis, dietary supplementation, and food fortification practices [10].

The frequent observation of low vitamin D levels, particularly in Africa and the Middle East, where abundant daily sunshine is available, may be explained by the traditional dress and avoidance of direct sunlight exposure, and multiple dietary factors as a result of specific cultural beliefs in these regions [15,45]. In Ethiopia, national or population data regarding vitamin D status in Ethiopia are non-existent, and, consequently, information on vitamin D is based on community- and health-facility-based surveys. The Ethiopian Food, Medicine and Health Care Administration and Control Authority (FMHACA) developed a directive for the import and wholesale of food supplements [46]. However, a vitamin D supplementation guideline for either the general population or groups at risk of vitamin D deficiency is unavailable. Estimating dietary intake and skin synthesis appears to be the major challenge for setting requirements. This is probably because sunshine is abundant in Ethiopia and vitamin D deficiency is thought to be unlikely to occur. Previous studies in Ethiopia reported vitamin D deficiency among school children (42%) [20], healthy non-pregnant women (84.2%), [19] and HIV (SVDD 28%) and TB/HIV co-infected patients (SVDD 57%) [21] and pulmonary tuberculosis patients [47,48]. Several factors including duration of sun exposure, chronic illness such as tuberculosis and HIV, female gender, clothing (most females in Ethiopia cover their head and most of their upper body when going outside), old age, and urban residence have been implicated in vitamin D deficiency in Ethiopia [19,47].

The prevalence of low vitamin D levels in the current study population was significantly high: 91.9% for chemotherapy naïve vs. 78.6% for the tamoxifen group. Particularly, for new patients who are about to initiate chemotherapy, the burden of subsequent vitamin D deficiency during chemotherapy treatment is expected to be very high. Although vitamin D status during chemotherapy was not investigated in the present study, other previous studies confirmed that 25(OH)D_3_ levels drop considerably further in breast cancer patients on anti-tumor treatment [44,49]. The impact of chemotherapy on vitamin D levels could be severe, such that it may not even be corrected sufficiently by vitamin D supplementation. It was reported that supplementation of vitamin D3 (cholecalciferol), at 400 IU daily, was insufficient to correct chemotherapy-induced vitamin D deficiency in pre-menopausal women with breast cancer undergoing adjuvant chemotherapy [50]. In contrast, a recent study reported that *de novo* vitamin D use post-diagnosis of breast cancer was found to be associated with a reduction in breast-cancer-specific mortality [42].

A plausible mechanism for chemotherapy-induced vitamin D deficiency has been described in the literature. The anti-neoplastic drugs such as taxol are ligands for the pregnane X receptor (PXR) and thereby enhance the catabolism of 25(OH)D_3_ and 1,25(OH)_2_D_3_ [51]. Anticancer chemotherapies are also known to cause gastro-intestinal toxicity [52], which can lead to reduced absorption of vitamin D from the gut [44]. Thus, vitamin D deficiency would be expected in almost all breast cancer patients receiving chemotherapy, which could lead to a greater risk of not only bone-health-related problems, but also compromise clinical outcomes of breast cancer treatment. A recent meta-analysis revealed evidence of an association between higher blood 25(OH)D_3_ concentrations and better survival in patients with colorectal cancer [53], indicating better disease prognosis and survival outcome with high circulating vitamin D. 

In this study, significantly lower mean vitamin D levels were found in treatment-naïve breast cancer patients compared to those on tamoxifen. Our finding is in agreement with a previous study which demonstrated patients on tamoxifen therapy to have significantly increased serum 25(OH)D_3_ levels [54]. Estrogen and selective estrogen receptor modulators (SERMs) can modulate *1-alpha-hydroxylase* activity in the kidney and facilitate the synthesis of more 1,25(OH)_2_D_3_ [55]. 

The *VDR* polymorphisms have been extensively explored in breast cancer risk assessment studies and their possible significance in breast cancer has been inconclusive. The *BsmI bb* or *Bb* genotype but not *Fok*I was associated with increased risk of breast cancer among Iranian and Egyptian female breast cancer patients [32,33]. However, a meta-analysis report concluded that, in the Caucasian ethnic subgroup or general population, *VDR* polymorphisms (*Fok1, Bsm1, Taq1,* and *Apa1*) were not associated with risk of breast cancer [56]. 

We have observed that carriers of the *VDR Fok*I *GG* (*FF*) genotype (OR = 1.44, 95% CI 1.01–2.05) were associated with risk of breast cancer. However, no association was detected between homozygotes for the common *Fok*I *F* allele (*GG* genotype) and *ff* homozygotes (*p* > 0.05). The *FokI FF* allele together with other *VDR* polymorphisms has been shown to amplify breast cancer risk in a Caucasian population [57]. In contrast to our finding, the *VDR*-*Fok*I *f* allele has been associated with increased risk of breast cancer in Canadians [34] and African Americans [58]. On the other hand, another study observed that there was no association between the *Fok*I polymorphism and breast cancer risk in postmenopausal women [59]. 

An experimental study conducted to evaluate the functional differences between *Fok*I polymorphic alleles in breast cancer cell lines demonstrated that, in response to 1α,25(OH)_2_D_3_ treatments, cell growth was inhibited by 60% in *FF* cells and 28% in *ff* cells. The induction of the vitamin D target gene *CYP24A1* mRNA was 1.8-fold higher in *FF* cells than in *ff* cells. Estrogen receptor-α protein expression was also down-regulated by 62% in *FF* cells and 25% in *ff* cells [60]. These findings suggest that the *ff* genotype may play a role in amplifying aggressive breast cancer. Results of meta-analysis also support the association of *Fok*I *ff* with breast cancer risk [30]. In the present study, the allele frequencies of the *VDR* alleles did not show evidence of significant differences between controls and cases. Consequently, the result observed in our study could not be ascertained. Moreover, the control groups were recruited from the general population and may not truly be negative for breast cancer, as some of them may develop the disease in the future. However, the *Fok*I *FF* allele frequencies are overrepresented in the cases compared to those from the general population who were free of cancer during the study enrolment.

Although the precise reasons are unknown, studies have highlighted racial disparities in cancer susceptibility and disease progression. Breast cancer tends to be diagnosed at a more advanced stage among black women than whites and, subsequently, black women experience elevated breast cancer mortality [61]. American women of African ancestry are more likely to develop breast cancer at a younger age than those with European ancestry and are more likely to have tumors with aggressive characteristics [62]. Another study also showed that more African Americans had severe vitamin D deficiency (<25 nmol/L) than European Americans with the lowest levels among those with the highest African ancestry [63]. The same study also revealed that genetic variants in the vitamin D pathway have been related to higher prevalence of estrogen receptor (ER)-negative breast cancer in African-American women [63]. In the present study, larger proportions (63.2%, mean age 40.77) of breast cancer patients were 40 years of age or below. The inherited genetic variations in the *VDR* gene may contribute in part to susceptibility to cancer at a younger age. However, evidence of an association was not detected between breast cancer characteristics and vitamin D deficiency or *VDR* genotype.

The present study investigated the severity of vitamin D deficiency among breast cancer patients in a resource-limited setting from Africa. Moreover, the study revealed pertinent information regarding the relevance of *VDR* polymorphism in a population with diverse genetic composition which has not been previously examined. However, important limitations were also identified. The small sample size could have hampered the observation of any association between vitamin D deficiency and clinico-pathology of breast cancer in Ethiopia. In addition, we used an immunoassay technique to measure vitamin D level rather than the gold-standard liquid chromatography coupled to tandem mass spectrometry (LC/MS/MS). Consequently, due to patient-group-specific deviations, a lack of distinction between 25(OH)D_2_ and 25(OH)D_3_, and cross-reactivity of other vitamin D metabolites associated with the immunoassay technique, the result of vitamin D concentration could have been overestimated and misleading. Moreover, chemotherapy-induced vitamin D deficiency and the impact of low vitamin D on the clinical progression of breast cancer was not addressed. Routine laboratory testing is not done to detect the receptor status of the tumor in Ethiopia, and, consequently, association of low vitamin D level with receptor status was not possible. Moreover, data on disease progression and survival status were not incorporated to associate them with vitamin D level and *VDR* polymorphism.

## 5. Conclusions

In conclusion, we report a high prevalence of vitamin D deficiency in female Ethiopian breast cancer patients. Treatment-naïve patients had low levels of vitamin D compared to patients on tamoxifen. In addition, the *Fok*I *GG* genotype appears to confer an increased risk of breast cancer in Ethiopian women. Further study is recommended to see the impact of chemotherapy on the vitamin D levels of breast cancer patients undergoing treatment and the impact of vitamin D deficiency on the disease progression and clinical outcome. We recommend supplementation of vitamin D and also urge further study to set the optimum dose of vitamin D for Ethiopian breast cancer patients.

## Figures and Tables

**Table 1:** Socio-demographic, clinical, and laboratory parameters and tumor characteristics of patient participants at baseline.

Parameter		Value
Socio-demographics		
Age (years, mean ± SD)**^♣^**		40.77 ± 10.79
BSA (m^2^, mean ± SD)		1.61 ± 0.19
BMI (Kg/m^2^, mean ± SD)		23.91 ± 4.61
Baseline laboratory results		
WBC (10^3^/mm^3^; median, IQR)		6.67 (2.74)
ANC (10^3^/mm^3^; median, IQR) Hgb (gm/dL; median, IQR)		3.59 (2.12) 13.9 (1.8)
HCT (%; median, IQR)		41.35 (4.48)
PLT (10^3^/mm^3^; median, IQR)		295.5 (105)
ALT (U/L; median, IQR)		18 (14)
AST (U/L; median, IQR)		24 (11)
ALP (U/L; median, IQR)		214 (141)
SCr (mean ± SD)		0.91 ± 0.18
BUN (median; IQR)		18 + 10
Tumor characteristics		*N*, %
Site of tumor	Left	200 (51.7)
	Right	177 (45.7)
	Bilateral	10 (2.6)
Histologic type of tumor	Ductal	332 (84.7)
	Lobular	17 (4.3)
	Mixed	4 (1)
	Other	39 (10)
Degree of differentiation	Well differentiated	33 (13.9)
	Moderately differentiated	116 (48.9)
	Poorly differentiated	88 (37.1)
Lymph node involvement	Negative	52 (16.7)
	Positive	259 (83.3)
Distant metastatic site	No known distant metastasis	63 (19.1)
	Bone, skin, or lung only	189 (57.3)
	Liver, CNS, lung + other organs	78 (23.6)

**^♣^** ALP alkaline phosphatase, ALT alanine aminotransferase, ANC absolute neutrophil count, AST aspartate aminotransferase, BUN, Blood urea nitrogen, BSA body surface area, Hgb hemoglobin, HCT hematocrit, IQR inter quartile range, PLT platelet count, SCr Serum creatinine, SD standard deviation, WBC white blood cell count.

**Table 2:** Comparison of the genotype and allele frequency distribution between breast cancer patients and healthy controls.

SNP	Genotype	Genotype Frequency by Group, *N* (%)			Allele Frequency by Group, *N* (%)			
		Cases	Controls	*p*-Value	Allele	Cases	Controls	*p*-Value
rs7975232 (*Apa*I, A > C)	AA	145 (37.5)	84 (43.7)	0.34	A	474 (61.2)	249 (64.8)	0.23
	AC	184 (47.5)	81 (42.2)		C	300 (38.8)	135 (35.2)	
	CC	58 (15)	27 (14.1)					
rs2228570 (*Fok*I, T > C)	AA	23 (5.9)	12 (6.4)	0.12	A	168 (21.5)	98 (26.2)	0.078
	AG	122 (31.3)	74 (39.6)		G	612 (78.5)	276 (73.8)	
	GG	245 (62.8)	101 (54)					
rs731236 (*Taq*I, T > C)	AA	149 (38.3)	74 (38.3)	0.33	A	481 (61.8)	230 (59.6)	0.46
	AG	183 (47)	82 (42.5)		G	297 (38.2)	156 (40.4)	
	GG	57 (14.7)	37 (19.2)					

**Table 3:** Association of *VDR* polymorphisms and breast cancer risk.

Chi-square test	**Gene**	**Genotype**	**Presence of Cancer**		***p*-Value**
			**Yes**	**No**	
	*rs7975232* (*Apa*I, A > C)	AA + AC	329 (66.6)	165 (33.4)	0.77
		CC	58 (68.2)	27 (31.8)	

	*rs2228570* (*Fok*I, T > C)	AA + AG	145 (62.8)	86 (37.2)	0.04
		GG	245 (70.8)	101 (29.2)	

	*rs731236* (*Taq*I, T > C)	AA + AG	332 (68)	156 (32)	0.16
		GG	57 (60.6)	37 (39.4)	

Logistic regression		**Univariate Analysis**		**Multivariate Analysis**	
		**^‡^ OR (95% CI)**	***p*-Value**	**OR (95% CI)**	***p*-Value**
	*rs2228570* (*Fok*I, T > C)				0.04
	AG + AA	1	1	1	
	GG	1.44 (1.01–2.05)	0.044	1.44 (1.01–2.06)	
	*rs731236* (*Taq*I, T > C)				-
	AG + AA	1		-	
	GG	0.72 (0.469–1.14)	0.164	-	

^‡^ OR odds ratio, CI confidence interval.

**Table 4:** Vitamin D status on the basis of plasma 25(OH)D_3_ concentration in chemotherapy-naïve patients and in those on tamoxifen adjuvant therapy.

Vitamin D Status ^♣^	Chemotherapy Naïve	Tamoxifen Group	*p*-Value
**SVDD (<25 nmol/L)**	46 (41.1%)	10 (11.2%)	<0.001
**VDD (25–50 nmol/L)**	56 (50%)	60 (67.4%)	
**Insufficient (51–72.5 nmol/L)**	9 (8%)	12 (14.6%)	
**Normal (72.5–250 nmol/L)**	1 (0.9%)	6 (6.7%)	

^♣^ SVDD, severe vitamin D deficiency; VDD, vitamin D deficiency.
